# Role of Contrast-Enhanced Ultrasound in the Characterization of Focal Splenic Lesions: A Systematic Review

**DOI:** 10.7759/cureus.113360

**Published:** 2026-07-25

**Authors:** Omar Amgad Elassra, Shashwat Shetty, Aakash Hans, Osama Ali, Saad Ali, Hira Khan, Mohit H Buch

**Affiliations:** 1 Medicine, Ain Shams University, Cairo, EGY; 2 Orthopaedics, Hillingdon Hospital, Uxbridge, GBR; 3 Research, Henry Ford Health System, Detroit, USA; 4 Internal Medicine, White River Health System, Batesville, USA; 5 Emergency Department, University Hospital Limerick, Limerick, IRL; 6 Emergency Department, Nishtar Hospital Multan, Multan, PAK; 7 Medicine, Jinnah Sindh Medical University, Karachi, PAK; 8 Radiology, Advanced Superspeciality Hospital, Gandhinagar, IND

**Keywords:** ceus, contrast-enhanced ultrasound, diagnostic imaging, focal splenic lesions, spleen

## Abstract

Contrast-enhanced ultrasound (CEUS) is an emerging imaging modality for the characterization of focal splenic lesions, offering real-time evaluation of microvascular perfusion without ionizing radiation. This systematic review assessed the diagnostic performance of CEUS in differentiating benign and malignant splenic lesions using data from five included studies. Across the evidence base, most benign lesions demonstrated sustained or prolonged enhancement, whereas many malignant lesions showed early washout with hypoenhancement, although overlap between lesion types was reported, reflecting underlying vascular disorganization. Diagnostic performance was high, with reported sensitivity ranging from 90% to 98% and specificity from 85% to 95% in primary diagnostic studies. CEUS improved lesion characterization compared with conventional ultrasound and may reduce the need for additional CT or MRI in selected clinical scenarios. However, the evidence was limited by heterogeneity in study design, small sample sizes, and moderate-to-high risk of bias in several studies. CEUS represents a reliable adjunct imaging tool for splenic lesion evaluation, though further large-scale prospective studies are required for standardization and validation.

## Introduction and background

Incidental focal splenic lesions are increasingly detected during abdominal imaging performed for unrelated clinical indications [1]. These lesions encompass a wide pathological spectrum, ranging from benign entities such as cysts, hemangiomas, hamartomas, and infarcts to malignant conditions, including lymphoma and metastatic disease [2]. Accurate differentiation between benign and malignant splenic lesions is clinically important because it directly influences patient management decisions, including the need for surveillance, further cross-sectional imaging, biopsy, or surgical intervention. Conventional ultrasound is commonly used as an initial imaging modality due to its wide availability, low cost, and lack of ionizing radiation [3]. However, its diagnostic performance is limited by overlap in the sonographic appearance of benign and malignant lesions, resulting in reduced specificity. Cross-sectional imaging modalities such as contrast-enhanced computed tomography (CT) and magnetic resonance imaging (MRI) offer improved anatomical resolution and better lesion detection, yet they may still demonstrate indeterminate findings in certain cases. Additionally, CT exposes patients to ionizing radiation, while MRI may be limited by availability, cost, and contraindications such as implanted devices or patient intolerance [4].

Contrast-enhanced ultrasound (CEUS) has emerged as an advanced imaging technique that enables real-time evaluation of microvascular perfusion using intravascular microbubble contrast agents [5]. Unlike CT and MRI, CEUS provides dynamic assessment of arterial, portal, and late phase enhancement without radiation exposure or nephrotoxic contrast agents. Characteristic enhancement patterns, including sustained enhancement in benign lesions and early washout with hypoenhancement in malignant lesions, have been increasingly recognized as valuable diagnostic markers that improve lesion characterization. Despite increasing evidence supporting the utility of CEUS in the evaluation of focal splenic lesions, there remains variability in reported diagnostic accuracy, imaging interpretation criteria, and clinical application across published studies. CT and MRI remain the principal cross-sectional imaging modalities for characterization of focal splenic lesions. CEUS provides complementary real-time assessment of lesion vascularity and may be particularly useful when MRI is contraindicated, when avoidance of ionizing radiation is preferred, or when further characterization of indeterminate lesions is required. Despite encouraging results, published evidence remains heterogeneous, with variations in study design, patient selection, imaging protocols, and interpretation criteria.

The primary aim of this study is to evaluate the diagnostic accuracy of CEUS in the characterization and differentiation of benign and malignant focal splenic lesions. The secondary aim is to describe the characteristic enhancement patterns of different splenic lesions on CEUS and to compare these findings with conventional imaging modalities such as ultrasound, CT, and MRI, as well as histopathological correlation where available. Additionally, this review aims to assess the clinical impact of CEUS in reducing the need for further imaging or invasive diagnostic procedures and to evaluate the overall methodological quality and risk of bias of the available literature on this topic.

## Review

Materials and methods

Search Strategy

A comprehensive and systematic literature search was conducted to identify all relevant studies evaluating the role of CEUS in the characterization of focal splenic lesions. The review was performed in accordance with the PRISMA 2020 guidelines to ensure transparency and reproducibility [6]. The search was carried out across four electronic databases: PubMed, Embase, Scopus, and the Cochrane Library. The search period covered all available literature from database inception to 1st May 2026. No lower date restriction was applied to capture all historical and contemporary evidence on CEUS applications in splenic imaging. A combination of Medical Subject Headings (MeSH) and free-text keywords was used. The search strategy included terms such as: “contrast-enhanced ultrasound”, “CEUS”, “splenic lesion”, “focal splenic mass”, “splenic tumour”, and “spleen imaging”. These terms were combined using Boolean operators “AND” and “OR” to improve the sensitivity and specificity of the search. An example of the search string used in PubMed was: (“contrast-enhanced ultrasound” OR “CEUS”) AND (“spleen” OR “splenic lesion” OR “focal splenic mass” OR “splenic tumour”). Similar strategies were adapted for other databases based on their indexing systems. All retrieved records were exported into reference management software, and duplicate entries were removed prior to screening. Two-stage screening was performed, consisting of title and abstract review followed by full-text assessment of eligible studies. Only studies published in the English language and involving human imaging data were included, although veterinary studies were considered for comparative imaging pattern analysis where relevant.

Eligibility Criteria

Eligibility criteria were defined according to the PICO (Population, Intervention, Comparator, and Outcomes) framework [7]. The population (P) included patients with focal splenic lesions found on imaging, including benign lesions such as cysts, hemangiomas, hamartomas, infarcts, and abscesses, as well as malignant lesions, including lymphoma and metastatic disease. Studies involving veterinary populations were also considered when they provided relevant data on CEUS enhancement characteristics and histopathological correlation. The intervention (I) was CEUS performed using second-generation microbubble contrast agents for the characterization of focal splenic lesions. The comparator (C) included conventional B-mode ultrasound, contrast-enhanced CT, MRI, histopathological examination, surgical findings, or clinical and radiological follow-up where tissue diagnosis was unavailable. The outcomes (O) of interest included diagnostic accuracy for differentiating benign from malignant lesions, enhancement and washout characteristics on CEUS, lesion vascularity patterns, diagnostic confidence, correlation with reference standards, and impact on clinical decision-making. Studies were eligible if they were original diagnostic accuracy studies, prospective or retrospective observational studies, narrative reviews, pictorial reviews, or educational reviews that provided clinically relevant information about CEUS findings in focal splenic lesions. Only studies published in English and available in full text were considered. Studies were excluded if they were case reports, conference abstracts, editorials, letters to the editor, studies evaluating traumatic splenic injury without focal lesions, studies not involving CEUS, or articles lacking sufficient imaging or outcome data. Veterinary studies were included. Studies with significant diagnostic relevance and histopathological correlation were kept for comparative analysis of CEUS enhancement patterns. Human studies formed the primary evidence base for this systematic review. Veterinary studies were not included in the diagnostic accuracy synthesis but were considered only as supplementary evidence describing enhancement patterns where histopathological correlation provided useful mechanistic insight.

Study Selection

Study selection was conducted according to the PRISMA 2020 guidelines [6]. All records identified through database searching were imported into reference management software, and duplicate citations were removed prior to screening. The remaining studies underwent title and abstract screening to assess relevance to the role of CEUS in the characterization of focal splenic lesions. Full-text articles of potentially eligible studies were then retrieved and evaluated against the predefined inclusion and exclusion criteria based on the PICO framework. Studies assessing CEUS in benign and malignant focal splenic lesions and reporting relevant imaging findings, diagnostic performance, or clinical outcomes were considered for inclusion. Case reports, editorials, conference abstracts, studies unrelated to splenic lesions, and articles lacking sufficient data were excluded. Although animal studies were generally excluded, veterinary studies with significant diagnostic relevance and histopathological correlation were considered where appropriate. Following full-text assessment, five studies fulfilled the eligibility criteria and were included in the final qualitative synthesis, with the study selection process documented using a PRISMA flow diagram.

Data Extraction

Data extraction was performed using a standardized data collection form. Information extracted from each study included author and year of publication, study design, sample size, population characteristics, and lesion types evaluated. Details regarding the CEUS protocol, contrast agent used, comparator modalities, and reference standards were also recorded. Radiological findings, including enhancement and washout patterns, diagnostic performance measures, and reported clinical outcomes, were systematically collected. Any discrepancies in data extraction were resolved through discussion and consensus.

Risk of Bias Assessment

The methodological quality of the included studies was assessed using the QUADAS 2 (Quality Assessment of Diagnostic Accuracy Studies 2) tool for diagnostic accuracy studies and the SANRA (Scale for the Assessment of Narrative Review Articles) checklist for review-based articles [8,9]. Assessment focused on patient selection, index test interpretation, reference standard, and flow and timing of investigations. Each study was categorized as having a low, moderate, or high risk of bias based on predefined criteria. Diagnostic studies generally demonstrated moderate risk of bias due to retrospective design and limited sample size, whereas narrative and pictorial reviews were considered at higher risk because of their non-systematic methodology. The results of the risk of bias assessment are presented in a dedicated summary table.

Data Synthesis

A data synthesis was performed due to heterogeneity in study designs, populations, imaging protocols, and outcome reporting. The included studies comprised retrospective diagnostic studies, a prospective veterinary study, and narrative and pictorial reviews, which precluded quantitative meta-analysis. Findings were grouped according to lesion type, CEUS enhancement patterns, and diagnostic performance where available. Consistent radiological trends were identified across studies, with benign lesions demonstrating sustained or isoenhancement and malignant lesions showing hypoenhancement with early washout.

Results

Study Selection Process

**Figure 1:**
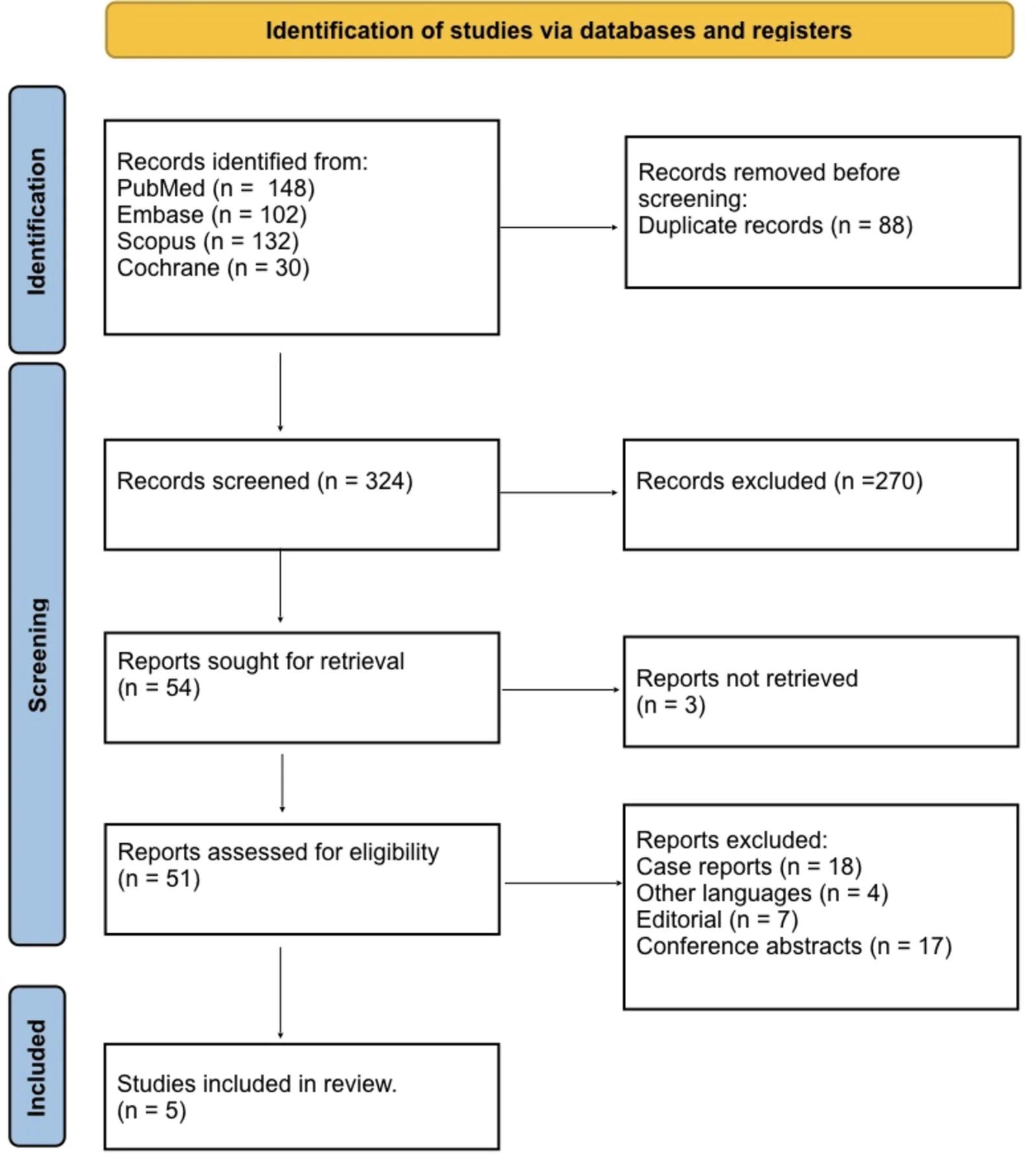
PRISMA 2020 flow diagram.

The study selection process was conducted in accordance with PRISMA guidelines. A total of 412 records were identified through database searching (n = 412), including PubMed (n = 148), Embase (n = 102), Scopus (n = 132), and the Cochrane Library (n = 30). After removal of duplicate records (n = 88), the remaining studies were screened based on titles and abstracts (n = 324), resulting in exclusion of irrelevant studies (n = 270). Full-text articles were then sought for retrieval (n = 54), of which some were not retrieved (n = 3). The remaining full-text articles were assessed for eligibility (n = 51), and studies were excluded for the following reasons: case reports (n = 18), other languages (n = 4), editorial articles (n = 7), and conference abstracts (n = 17). Ultimately, five studies meeting the inclusion criteria were included in the final systematic review (Figure 1).

Characteristics of the Selected Studies

The characteristics of the selected studies are presented in Table 1. García Barquín et al. (2025) evaluated patients with focal splenic lesions using CEUS compared with CT, MRI, ultrasound, histopathology, and follow-up imaging, demonstrating that malignant lesions typically show abnormal microvascularity with hypoenhancement and washout, while benign lesions show sustained enhancement, with reported sensitivity of 90-98% and specificity of 85-95%, contributing to reduced need for cross-sectional imaging [10]. Rossi et al. (2008) assessed 35 dogs with splenic masses using CEUS compared with histopathology, showing that malignant lesions demonstrate heterogeneous vascularity with rapid washout patterns, whereas benign lesions show more homogeneous enhancement, with sensitivity of 92-95% and specificity of 85-90% supporting preoperative lesion classification [11]. Yang et al. (2021) studied 123 patients using CEUS compared with conventional ultrasound and histopathology, reporting that malignant lesions exhibit irregular vascularity with early washout and hypoenhancement, while benign lesions show persistent enhancement, with high diagnostic accuracy (sensitivity = 94.6%, specificity = 91.3%, area under the curve (AUC) = 0.94) and improved diagnostic confidence [12]. Harvey et al. (2008) provided a literature-based review describing CEUS imaging patterns across splenic lesions, highlighting microvascular differences between benign and malignant pathology and establishing characteristic enhancement patterns without reporting quantitative diagnostic performance [13]. Zavariz et al. (2017) performed a case-based imaging review comparing CEUS findings with CT, MRI, and pathology, demonstrating that benign lesions show arterial enhancement with persistent late-phase filling, while malignant lesions show early washout, with reported sensitivity of 88-95% based on literature synthesis and improved lesion recognition in clinical practice [14].

**Table 1:** Characteristics of the selected studies. CEUS: contrast-enhanced ultrasound; CT: computed tomography; MRI: magnetic resonance imaging; US: ultrasound; AUC: area under the curve.

Authors & year	Population (P)	Intervention (I)	Comparator (C)	Outcomes (O)	Pathophysiological findings	Anatomical impact	CEUS findings	Diagnostic performance	Clinical Impact
García Barquín et al., 2025 [10]	Patients with focal splenic lesions	CEUS	CT, MRI, US, histopathology	Lesion characterization	Malignancy shows abnormal microvascularity with washout	Whole spleen focal lesions	Benign: sustained enhancement; malignant: hypoenhancement + washout	Sensitivity = 90-98%, specificity = 85-95%	Reduced need for cross-sectional imaging
Rossi et al., 2008 [11]	35 dogs	CEUS	Histopathology	Lesion differentiation	Malignant lesions show heterogeneous vascularity	Splenic masses	Rapid washout in malignancy	Sensitivity = 92-95%, specificity = 85-90%	Preoperative lesion classification
Yang et al., 2021 [12]	123 patients	CEUS	US + histopathology	Diagnostic accuracy	Irregular vascularity in malignancy	Focal splenic lesions	Early washout + hypoenhancement in malignancy	Sensitivity = 94.6%, specificity = 91.3%, AUC = 0.94	Improved diagnostic confidence
Harvey et al., 2008 [13]	Literature review	CEUS	CT/MRI/US	Imaging patterns	Microvascular differences in lesions	Splenic lesions spectrum	Classic enhancement patterns described	Not reported	Foundational imaging framework
Zavariz et al., 2017 [14]	Case-based review	CEUS	CT/MRI/pathology	Imaging characterization	Vascular architecture-based classification	Benign and malignant lesions	Arterial + late washout patterns	88-95% sensitivity (literature-based)	Improved pattern recognition

Risk of Bias Assessment

The included studies demonstrated variable methodological quality, with diagnostic studies showing moderate risk of bias primarily due to retrospective design, small sample size, and potential selection bias, as shown in Table 2. The veterinary study by Rossi et al. (2008) was also graded as moderate risk owing to limited direct applicability to human populations despite strong histopathological correlation [11]. In contrast, the narrative and pictorial reviews by García Barquín et al. (2025) [10], Harvey et al. (2008) [13], and Zavariz et al. (2017) [14] were all assessed as high risk of bias due to their non-systematic methodology and absence of quantitative analysis. The retrospective diagnostic study by Yang et al. (2021) demonstrated moderate risk of bias because of single-center design and possible selection bias [12]. These limitations highlight the heterogeneity and predominantly descriptive nature of the current evidence base on CEUS in focal splenic lesions.

**Table 2:** Risk bias assessment. QUADAS-2: Quality Assessment of Diagnostic Accuracy Studies 2; SANRA: Scale for the Assessment of Narrative Review Articles.

Study	Study design	Risk of bias tool	Risk of bias rating	Justification
García Barquín et al., 2025 [10]	Narrative review	SANRA	High	Non-systematic review, no protocol
Rossi et al., 2008 [11]	Prospective veterinary study	QUADAS-2	Moderate	Small sample, limited human applicability
Yang et al., 2021 [12]	Retrospective diagnostic study	QUADAS-2	Moderate	Single-center, selection bias possible
Harvey et al., 2008 [13]	Encyclopedic review	SANRA	High	No original data
Zavariz et al., 2017 [14]	Pictorial review	SANRA	High	Descriptive, no quantitative synthesis

CEUS Enhancement Patterns According to Lesion Type

Hemangiomas generally demonstrated peripheral or diffuse sustained enhancement throughout the late phase. Hamartomas commonly showed homogeneous enhancement with persistent enhancement. Splenic infarcts remained non-enhancing. Abscesses demonstrated variable enhancement with peripheral rim enhancement in some cases. Lymphoma frequently demonstrated early washout and hypoenhancement, although atypical appearances were reported. Metastases generally demonstrated early washout with hypoenhancement.

Discussion

CEUS has emerged as a valuable imaging modality for the characterization of focal splenic lesions, offering real-time assessment of microvascular perfusion. Across the included studies, CEUS consistently demonstrated a strong ability to differentiate benign from malignant lesions based on enhancement behavior. This was particularly evident in Yang et al. (2021), who reported high diagnostic performance with sensitivity of 94.6%, specificity of 91.3%, and AUC of 0.94, reinforcing its role as a reliable adjunct to conventional imaging [12]. Similarly, García Barquín et al. (2025) demonstrated pooled evidence supporting sensitivity of 90-98% and specificity of 85-95%, further confirming its diagnostic robustness across multiple imaging modalities [10]. The spectrum of focal splenic lesions evaluated across the included studies included cysts, hemangiomas, hamartomas, infarcts, abscesses, lymphoma, and metastases. Despite heterogeneity in study design, a consistent pattern of vascular behavior was observed. Most benign lesions tend to demonstrate sustained or isoenhancement reflecting preserved microvascular architecture, whereas malignant lesions showed hypoenhancement with early washout due to abnormal tumor neovascularity. This pattern was consistently described across both human and veterinary literature, including Rossi et al. (2008), who demonstrated rapid washout in malignant lesions in a histopathologically confirmed cohort [11], and Zavariz et al. (2017), who reinforced arterial enhancement with late washout as a key malignant feature [14].

In terms of diagnostic performance, CEUS showed consistently high accuracy across primary diagnostic studies. As shown in Table 1, Yang et al. (2021) demonstrated the highest reported accuracy among human studies, with excellent discrimination between benign and malignant lesions [12]. García Barquín et al. (2025) further supported these findings through a broader evidence synthesis, highlighting CEUS as a reliable modality capable of reducing the need for cross-sectional imaging in selected patients [10]. Compared with conventional ultrasound, CEUS provides superior lesion characterization by enabling dynamic vascular phase assessment, while CT and MRI remain complementary but may still yield indeterminate results in certain lesions. Across the included human studies, CEUS findings were compared against histopathology, CT, MRI, conventional ultrasound, or imaging follow-up. CEUS consistently improved characterization of indeterminate lesions compared with conventional ultrasound and showed good agreement with histopathology where available. CT and MRI remained important complementary imaging modalities, particularly in staging and comprehensive lesion assessment. CEUS may reduce the need for additional CT or MRI in selected patients after appropriate clinical assessment. Interpretation of CEUS findings should always consider lesion morphology, clinical presentation, laboratory findings, and complementary imaging because overlap exists between benign and malignant enhancement characteristics.

Despite these advantages, several limitations were identified across the included studies. Most studies demonstrated moderate-to-high risk of bias, particularly due to retrospective design, small sample sizes, and non-standardized imaging protocols. The veterinary study by Rossi et al. (2008) provided strong histopathological correlation but limited direct generalizability to human populations [11]. Review-based studies, including Harvey et al. (2008) and Zavariz et al. (2017), lacked systematic methodology and quantitative synthesis, limiting their strength in evidence-based conclusions [13,14]. Additionally, variability in contrast agents, scanning protocols, and interpretation criteria further contributes to heterogeneity in reported outcomes. Despite encouraging results, published evidence remains heterogeneous, with variations in study design, patient selection, imaging protocols, and interpretation criteria.

Future directions should focus on addressing these methodological limitations through large-scale multicenter prospective studies with standardized CEUS acquisition and interpretation protocols. Development of structured reporting systems for splenic lesions, similar to the Liver Imaging Reporting and Data System (LI-RADS) in hepatic imaging, may improve reproducibility and diagnostic confidence. Furthermore, integration of quantitative perfusion analysis and artificial intelligence-based imaging assessment may enhance lesion classification and reduce operator dependency.

## Conclusions

CEUS appears to be a promising adjunct imaging modality for characterization of focal splenic lesions, demonstrating strong diagnostic accuracy in distinguishing benign from malignant pathology based on characteristic enhancement patterns. It provides significant clinical advantages over conventional ultrasound by enabling real-time assessment of lesion vascularity and improving diagnostic confidence, while it may reduce the need for additional cross-sectional imaging in carefully selected clinical scenarios. However, the current evidence is limited by study heterogeneity, small sample sizes, and methodological constraints, highlighting the need for large multicenter prospective studies and standardized imaging criteria to fully establish its role in routine clinical practice. Further multicenter prospective studies using standardized acquisition protocols and reporting criteria are required before broader implementation into routine clinical practice.
